# Neonatal Azithromycin Administration and Growth during Infancy: A Randomized Controlled Trial

**DOI:** 10.4269/ajtmh.22-0763

**Published:** 2023-03-27

**Authors:** Ali Sie, Mamadou Bountogo, Alphonse Zakane, Guillaume Compaoré, Thierry Ouedraogo, Mamadou Ouattara, Elodie Lebas, Jessica Brogdon, Fanice Nyatigo, Kieran S. O’Brien, Travis C. Porco, Till Bärnighausen, Benjamin F. Arnold, Thomas M. Lietman, Catherine E. Oldenburg, for the NAITRE Study Team

**Affiliations:** 1 Centre de Recherche en Santé de Nouna, Burkina Faso;; 2 Francis I Proctor Foundation, University of California, San Francisco;; 3 Department of Ophthalmology, University of California, San Francisco;; 4 Department of Epidemiology & Biostatistics, University of California, San Francisco;; 5 Heidelberg Institute for Global Health, University of Heidelberg, Germany;; 6 Africa Health Research Institute, Somkhele, South Africa;; 7 Department of Global Health and Population, Harvard T.H. Chan School of Public Health, Boston, Massachusetts

## Abstract

Observational studies have linked early-life antibiotic exposure to increased risk of obesity in children in high income settings. We evaluated whether neonatal antibiotic exposure led to changes in infant growth at 6 months of age in Burkina Faso. Neonates aged 8 to 27 days of age who weighed at least 2,500 g at the time of enrollment were randomized in a 1:1 fashion to a single oral 20-mg/kg dose of azithromycin or equivalent volume of placebo from April 2019 through December 2020. Weight, length, and mid-upper-arm circumference (MUAC) were measured at baseline and 6 months of age. Growth outcomes, including weight gain in grams per day, length change in millimeters per day, and changes in weight-for-age Z-score (WAZ), weight-for-length Z-score (WLZ), length-for-age Z-score (LAZ), and MUAC were compared among neonates randomized to azithromycin compared with placebo. Among 21,832 neonates enrolled in the trial, median age at enrollment was 11 days, and 50% were female. We found no evidence of a difference in weight gain (mean difference −0.009 g/day, 95% confidence interval [CI]: −0.16 to 0.14, *P* = 0.90), length change (mean difference 0.003 mm/day, 95% CI: −0.002 to 0.007, *P* = 0.23), or WAZ (mean difference −0.005 SD, 95% CI: −0.03 to 0.02, *P* = 0.72), WLZ (mean difference −0.01 SD, 95% CI: −0.05 to 0.02, *P* = 0.39), LAZ (mean difference 0.01, 95% CI: −0.02 to 0.04, *P* = 0.47), or MUAC (mean difference 0.01 cm, 95% CI: −0.02 to 0.04, *P* = 0.49). These results do not suggest that azithromycin has growth-promoting properties in infants when administered during the neonatal period.

Trial registration: ClinicalTrials.gov NCT03682653.

## INTRODUCTION

Undernutrition during early infancy predisposes children to later growth failure, deficits in cognitive development, and mortality.^1^^,^^2^ Although most public health programs aiming to reduce undernutrition in children focus on children aged 6 to 59 months, linear growth faltering is present at birth and continues in the postnatal period, and new-onset wasting is highest from birth through 3 months of age.^3^^,^^4^ Targeting interventions early during the highest incidence period may be particularly beneficial for prevention of undernutrition and its sequelae.

Antibiotics are commonly used for growth promotion in food animals.^5^ Although the mechanism behind the growth-promoting effect of antibiotics in animals is unclear, it may be via treatment of pathogens present in animal feed that can inhibit nutrient absorption or may be treatment of pathogenic bacteria in the animals themselves.^6^ In children, antibiotics have been shown to increase weight gain in randomized controlled trials among children with established morbidity (e.g., HIV, severe acute malnutrition) in low-resource settings.^7^ Observational studies have suggested a link between early-life antibiotic exposure and obesity.^8^^–^^12^ These studies are limited by confounding by indication, and the role of antibiotics for promoting growth and any potential link to childhood obesity remains uncertain.^13^ A growth-promoting effect of antibiotics when administered during early infancy in settings with a high burden of undernutrition may be protective against adverse childhood outcomes, although it could also contribute to obesity in some settings. In settings with high exposure to enteric infections during infancy, antibiotics could be growth promoting if they treat clinical or subclinical infection.

Biannual mass azithromycin distribution to all children in a given community, regardless of whether they have clinical or subclinical infection, with a single oral 20-mg/kg dose has been shown to lead to reductions in all-cause childhood mortality in sub-Saharan Africa.^14^ The largest effects were in children aged 1 to 5 months, with an approximately 25% reduced risk of mortality.^14^^,^^15^ The precise mechanism behind this action is unknown but presumably involves a reduction in clinical or subclinical infections. One hypothesized mechanism for this reduction is via improvements in childhood growth and nutrition, either via treatment of subclinical infection or environmental enteric dysfunction and subsequently improved growth, or a direct growth-promoting effect of antibiotics. Increased weight gain in children receiving antibiotics without established infection could be protective against poor outcomes or reflective of a reduction in pathogen burden but could also predispose otherwise healthy children to obesity if antibiotics are growth promoting. Although antibiotics should not be withheld in cases with clear bacterial infection regardless of effects on weight gain, for presumptive or empiric treatment effects on weight gain could be considered in decision-making related to antibiotic use.

The NAITRE trial was a 1:1 randomized placebo-controlled trial designed to assess whether a single oral dose of azithromycin administered during the neonatal period reduces all-cause infant mortality in Burkina Faso. In this prespecified secondary analysis of the parent trial, we evaluated whether a single dose (20 mg/kg) of azithromycin, similar to that used in previous studies of azithromycin for prevention of childhood mortality, administered during the neonatal period (8 to 27 days of life) resulted in greater child growth compared with placebo among infants. We hypothesized that neonates receiving azithromycin would have greater weight gain and linear growth compared with those receiving placebo.

## METHODS

### Parent trial design.

Complete trial methods for the parent study have been previously published.^16^^,^^17^ The Nouveux-nés et Azithromycine: une Innovation dans le Traitement des Enfants (NAITRE) trial was a 1:1 randomized placebo-controlled trial designed to evaluate the efficacy of a single oral dose of 20 mg/kg azithromycin administered to neonates for the prevention of infant mortality. This prespecified secondary analysis of the trial aims to evaluate effects of azithromycin compared with placebo on growth endpoints. The analysis was prespecified in the trial’s statistical analysis plan (available with the primary study report).^16^^,^^17^

### Ethics.

The trial was reviewed and approved by the Comité d’Ethique pour la Recherche en Santé in Ouagadougou (Protocol #2018-10-123) and the Institutional Review Board at the University of California, San Francisco (Protocol #18-25027). Written informed consent was obtained from the caregiver of each enrolled infant. The trial was overseen by a Data and Safety Monitoring Committee (DSMC) that consisted of independent experts in pediatric infectious disease, randomized controlled trials, mass azithromycin distribution, bioethics, and biostatistics. The DSMC reviewed quarterly data reports in aggregate and met face-to-face once annually to ensure participant safety, review trial progress and adverse events, and recommend continuation or discontinuation of the trial. Safety monitoring including measurement of caregiver-reported adverse events at each study visit and active surveillance for infantile hypertrophic pyloric stenosis. Full details are reported in the trial’s primary endpoint report.^17^

### Study setting.

Participants were enrolled in 44 Centers de Santé et de Promotion Sociale (CSPS) in five regions of Burkina Faso. CSPSs are the first level of the government-run healthcare system and offer services such as antenatal care and vaccination visits. These services are free of charge for pregnant women and children under 5 years of age. Burkina Faso is located in the Sahel in West Africa and experiences highly seasonal rainfall, from approximately July through October. The rainy season coincides with the high malaria transmission season. During the rainy season, children aged 3 to 59 months in the study catchment areas are eligible for monthly seasonal malaria chemoprevention (SMC) with sulfadoxine-pyramethamine. Although children under 3 months of age are not eligible for SMC; infants enrolled in the present study who were between 3 and 6 months of age from July through October may therefore have received up to four doses of SMC that coincided with the follow-up period of the study.

### Recruitment and eligibility criteria.

Caregivers of newborns born at participating facilities and those bringing their children for routine vaccination days were informed of the study and how to participate. At participating facilities, BCG vaccination typically occurs during the weeks after birth rather than at birth. Those who were interested in the study were referred to the facility’s study nurse for formal eligibility assessment and enrollment. Neonates were eligible if they were between 8 and 27 days old at the time of enrollment, weight at least 2,500 g, had no known allergies to azalides or macrolides, were able to feed orally, and had no clinical signs of neonatal jaundice. Neonates who were too young or too small at their first evaluation could return for a second assessment once they had aged into the eligible age range or had grown for possible inclusion, provided they still met all eligibility criteria. Infants weighing less than 2,500 g at enrollment were excluded from the trial at the recommendation of the trial’s DSMC due to concerns that very small babies may be at increased risk of infantile hypertrophic pyloric stenosis.^16^^,^^17^

### Intervention and randomization.

Participants were randomized in a 1:1 fashion to a single oral 20-mg/kg dose of azithromycin or matching placebo (both provided by Pfizer, Inc, New York, NY) after enrollment and baseline assessments. Dosing was based on a weight measurement on a standard infant scale (ADE M112600 U Scale) and the study’s electronic data capture application automatically calculated the volume of a 20 mg/kg dose of azithromycin or equivalent volume of placebo. Study treatment was delivered by syringe, and all study treatments were directly observed and recorded in the study’s data capture application. The randomization sequence was generated by the trial’s unmasked data team without blocking or stratification using R (The R Foundation for Statistical Computing, Vienna, Austria).

### Masking and allocation concealment.

Masking was achieved via the use of a placebo that was identical in appearance, taste, and smell to the azithromycin. The formulation of the two medications was the same except for the active ingredient. To facilitate both masking and allocation concealment, study medication bottles were labeled with one of eight treatment letters, with four letters randomly assigned to correspond to azithromycin and four to placebo. Medication labels were identical except for the randomly assigned treatment letter. Each unique study identification number that was assigned to participants after enrollment was randomized to one of the eight treatment letters. The treatment letter was only revelated after enrollment and baseline procedures were complete, and the child was treated with a medication bottle labeled with the corresponding treatment letter. Study participants, caregivers, staff, and investigators were masked to treatment allocation.

### Follow-up.

All infants enrolled in the parent trial were followed at 21 days after enrollment and at 3, 6, and 12 months of life. Anthropometric measurements were collected at enrollment (age 8 to 27 days) and 6 months of life, and thus all data included in this report are from the enrollment and 6 months of age time points.

### Anthropometric assessments.

All infants enrolled in the trial were eligible for anthropometric measurement at enrollment and 6 months. At both timepoints, children were weighed with a standard infant scale, length was measured using a ShorrBoard (Weight and Measure, LLC, Olney, MD) and mid-upper-arm circumference (MUAC) was measured using a standard MUAC tape (Weight and Measure, LLC, Olney, MD). Length measurements were collected in triplicate and the median was used for all analyses. Children with MUAC measurements <11.5 cm at the 6-month study visit were referred to the health facility’s nutritional program for assessment of acute malnutrition. The infant scale was standardized each morning before measuring any infants using a 2-kg test weight. Any scale that did not pass calibration was replaced. All study personnel underwent a 2-day didactic and practical training for anthropometric measurements before the beginning of the study. Study personnel conducted monthly monitoring visits to each enrollment site to observe and provide refresher training as needed and at least once per year. Study data underwent weekly monitoring and outlying values were flagged, and study supervision visits were made to sites if there were concerns about outlying values.

### Anthropometric outcomes.

Weight gain in grams per day, and length change in millimeters per day were calculated at 6 months of age since baseline. Weight-for-age Z-score (WAZ), length-for-age Z-score (LAZ), and weight-for-length Z-score (WLZ) were calculated separately at baseline and 6 months per 2006 WHO standards.^18^ All outcome measurements were at 6 months of age with a window of ±6 weeks of age for outcome assessment (141–225 days). WAZ scores outside of the range of −6 to +5 SD, LAZ −6 to +6 SD, and WLZ −5 to +5 SD were considered outliers per 2006 WHO Child Growth Standards and not included in analyses.

### Sample size for anthropometric endpoints.

For this analysis, we estimated the minimum detectable effect on growth outcomes given the sample size that was determined for the trial’s primary endpoint, mortality by age 6 months. By the 6-month visit, there were 9,475 children measured in the azithromycin arm and 9,627 in the control arm. Assuming 9,475 children per group with 90% power with a two-sided alpha of 5%, we estimated the minimum detectable effect for LAZ, WLZ, and WAZ given the study’s empirical standard deviation for each outcome (LAZ SD = 1.15, WLZ SD = 1.19, WAZ SD = 1.05) adjusted for the correlation with baseline (LAZ r = 0.37, WLZ r = 0.20, WAZ r = 0.34). The minimum detectable effect sizes for this trial were LAZ = 0.05, WLZ = 0.06, and WAZ = 0.05.

### Statistical methods.

Weight gain in grams per day was calculated by calculating the difference in 6-month weight from baseline weight and dividing it by the number of days from enrollment to the 6-month study visit. Similarly, length change in millimeters per day was calculated as the difference in length at the two visits, divided by the number of days between visits. Unadjusted linear regression models were used to estimate the difference in grams per day and millimeters per day in neonates receiving azithromycin compared with placebo. Linear regression models adjusted for each baseline measure were used to estimate the difference in weight, length, WAZ, LAZ, WLZ, and MUAC at 6 months of age in infants randomized to azithromycin compared with placebo. Prespecified subgroup analyses for the primary outcome included age of enrollment by week (2, 3, or 4 weeks of age), sex (male or female), season of enrollment (rainy, defined as June through October, or dry), and urbanicity (urban, periurban, or rural). Subgroup analyses were similar to main analyses but included an interaction term for the subgroup by treatment arm for estimation of interaction on the additive scale and the main effect of the subgroup term. Models evaluating the effect of azithromycin on anthropometric endpoints were not adjusted for any covariates with the exception of the baseline measure for each outcome to improve statistical power. Because the intervention (azithromycin or placebo) was randomized, by definition there is no confounding (defined as a common cause of exposure and outcome), and no adjustment for confounders is necessary. Our prespecified analysis plan did not include adjustment for any covariates other than baseline measures, and all models followed the prespecified statistical analysis plan. All analyses were two-sided, and an alpha of < 0.05 was considered statistically significant. All analyses were conducted in R.

## RESULTS

**Figure 1.**
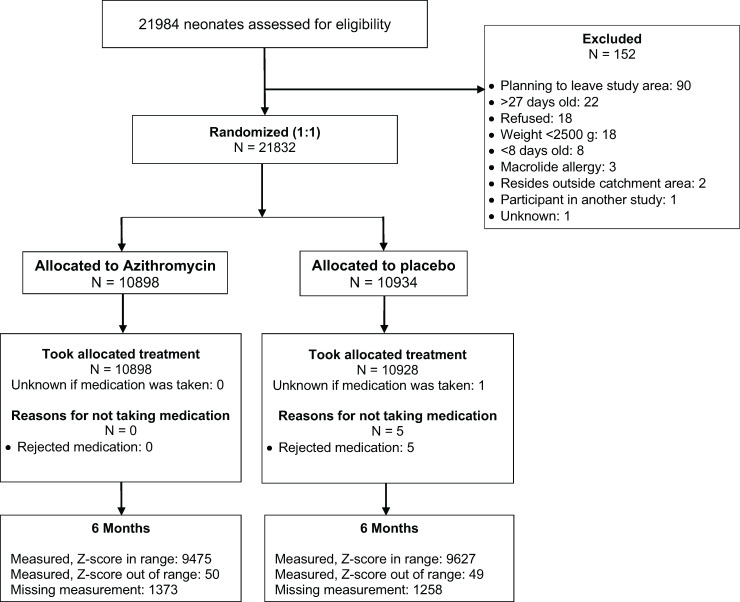
Screening, randomization, and follow-up of participants.

Of 21,832 enrolled neonates enrolled between April 2019 and December 2020, 10,898 were randomized to azithromycin and 10,934 were randomized to placebo (Figure 1). At enrollment, anthropometric measurements were available for 10.898 participants in the azithromycin arm and 10,933 participants in the placebo arm. Participants were a median age of 11 days at enrollment in each group and 49.7% were female. There were no notable differences in baseline characteristics between treatment groups (Table 1). Median birthweight was 3,000 g in each group. At enrollment, 7.3% of infants in the azithromycin group and 6.9% in placebo group were underweight (WAZ < −2) and 7.3% in both groups were stunted (LAZ < −2). At 6 months, valid anthropometric measurements were available for 9,475 participants in the azithromycin arm and 9,627 participants in the placebo arm.

**Table 1:** Baseline characteristics by randomized treatment group

Characteristic	Azithromycin (*n* = 10,898)	Placebo (*n* = 10,934)	Overall (*n* = 21,832)
Age, days, median (IQR)	11 (9–15)	11 (9–14)	11 (9–14)
Sex			
Female	5,413 (49.7%)	5,431 (49.7%)	10,844 (49.7%)
Male	5,485 (50.3%)	5,503 (50.3%)	10,988 (50.3%)
Region			
Center	919 (8.4%)	951 (8.7%)	1,870 (8.6%)
Boucle du Mouhoun	1,299 (11.9%)	1,329 (12.2%)	2,628 (12.0%)
Cascade	2,009 (18.4%)	1,977 (18.1%)	3,986 (18.3%)
Center Ouest	1,217 (11.2%)	1,211 (11.1%)	2,428 (11.1%)
Hauts-Bassins	5,454 (50.0%)	5,465 (50.0%)	10,919 (50.0%)
Urban dwelling			
Urban	8,960 (82.2%)	9,055 (82.8%)	18,015 (82.5%)
Rural	1,932 (17.7%)	1,869 (17.1%	3,801 (17.4%)
Birthweight, g, median (IQR)	3,000 (2,700–3,250)	3,000 (2,700–3,260)	3,000 (2,700–3,250)
Weight at enrollment, g, median (IQR)	3,300 (2,980–3,620)	3,300 (2,990–3,620)	3,300 (2,990–3,620)
Length at enrollment, cm, median (IQR)	50.4 (49.3–51.9)	50.5 (49.3–52.0)	50.5 (49.3–51.9)
WLZ, mean (SD)	−0.63 (1.3)	−0.65 (1.3)	−0.64 (1.3)
WAZ, mean (SD)	−0.62 (0.9)	−0.61 (0.9)	−0.62 (0.9)
LAZ, mean (SD)	−0.54 (1.1)	−0.50 (1.1)	−0.52 (1.1)
MUAC, median (IQR)	10.9 (10.0–11.5)	11.0 (10.0–11.5)	11.0 (10.0–11.5)
Mother’s age, median (IQR)	25 (21–30)	25 (21–30)	25 (21–30)
Mother’s education			
None	5,910 (54.2%)	6,029 (55.1%)	11,939 (54.7%)
Primary	1,990 (18.3%)	1,978 (18.1%)	3,968 (18.2%)
Secondary or above	2,997 (27.5%)	2,923 (26.7%)	5,920 (27.1%)
No. children in household, median (IQR)	1 (0–3)	1 (0–3)	1 (0–3)
Pregnancy type			
Singleton	10,702 (98.2%)	10,753 (98.3%)	21,455 (98.3%)
Multiple	195 (1.8%)	177 (1.6%)	372 (1.7%)
No. of antenatal visits, median (IQR)	4 (3–5)	4 (3–5)	4 (3–5)
Initiation of breastfeeding			
Immediate	10,320 (94.7%)	10,341 (94.6%)	20,661 (94.6%)
Delayed	566 (5.2%)	574 (5.2%)	1,140 (5.2%)
Not breastfeeding	11 (0.1%)	15 (0.1%)	26 (0.1%)

IQR = interquartile range; LAZ = length-for-age Z-score; MUAC = mid-upper-arm circumference; WAZ = weight-for-age Z-score; WLZ = weight-for-length Z-score.

At 6 months of age, mean weight gain was 23.2 g/day in infants receiving azithromycin compared with 23.3 g/day in those receiving placebo, corresponding to a mean difference of −0.009 g/day (95% confidence interval [CI]: −0.16 to 0.14 g/day, *P* = 0.90; Table 2). Mean length change was 0.9 mm/day in both the azithromycin and placebo groups (mean difference 0.003 mm/day, 95% CI: −0.002 to 0.007, *P* = 0.23). There was no evidence of a difference in WAZ, WLZ, LAZ, or MUAC between groups (Table 2).

**Table 2:** Anthropometric outcomes by randomized treatment group among infants 6 months of age who received azithromycin or placebo between 8 and 27 days of age

Anthropometric measurement	*N* *	Azithromycin	Placebo	Mean difference (95% CI)	*P* value
		Mean (SD)	Mean (SD)		
Weight gain, g/day	19,101	23.2 (5.3)	23.3 (5.4)	−0.009	0.90
				(−0.16–0.14)	
Length change, mm/day	19,098	0.9 (0.2)	0.9 (0.2)	0.003	0.23
				(−0.002–0.007)	
Weight, kg†	19,101	7.3 (0.9)	7.3 (0.9)	−0.009	0.48
				(−0.03–0.02)	
Length, cm†	19,098	65.8 (2.7)	65.8 (2.6)	0.007	0.85
				(−0.06–0.08)	
WAZ†	19,101	−0.44 (1.1)	−0.43 (1.1)	−0.005	0.72
				(−0.03–0.02)	
WLZ†	19,051	−0.13 (1.2)	−0.12 (1.2)	−0.01	0.39
				(−0.05–0.02)	
LAZ†	19,098	−0.46 (1.2)	−0.46 (1.2)	0.01	0.47
				(−0.02–0.04)	
MUAC, cm†‡	18,430	14.1 (1.2)	14.1 (1.1)	0.01	0.49
				(−0.02–0.04)	

CI = confidence interval; LAZ = length-for-age Z-score; MUAC = mid-upper-arm circumference; WAZ = weight-for-age Z-score; WLZ = weight-for-length Z-score.

* *N*s in analysis vary slightly due to missingness at baseline (*N* = 1 missing weight measure at baseline, *N* = 4 missing length measures at baseline, *N* = 51 missing WHZ measures at baseline, *N* = 82 missing MUAC measures at baseline).

† Adjusted for baseline value of the outcome.

‡ MUAC measurements available for 18,512 children at 6 months.

**Figure 2.**
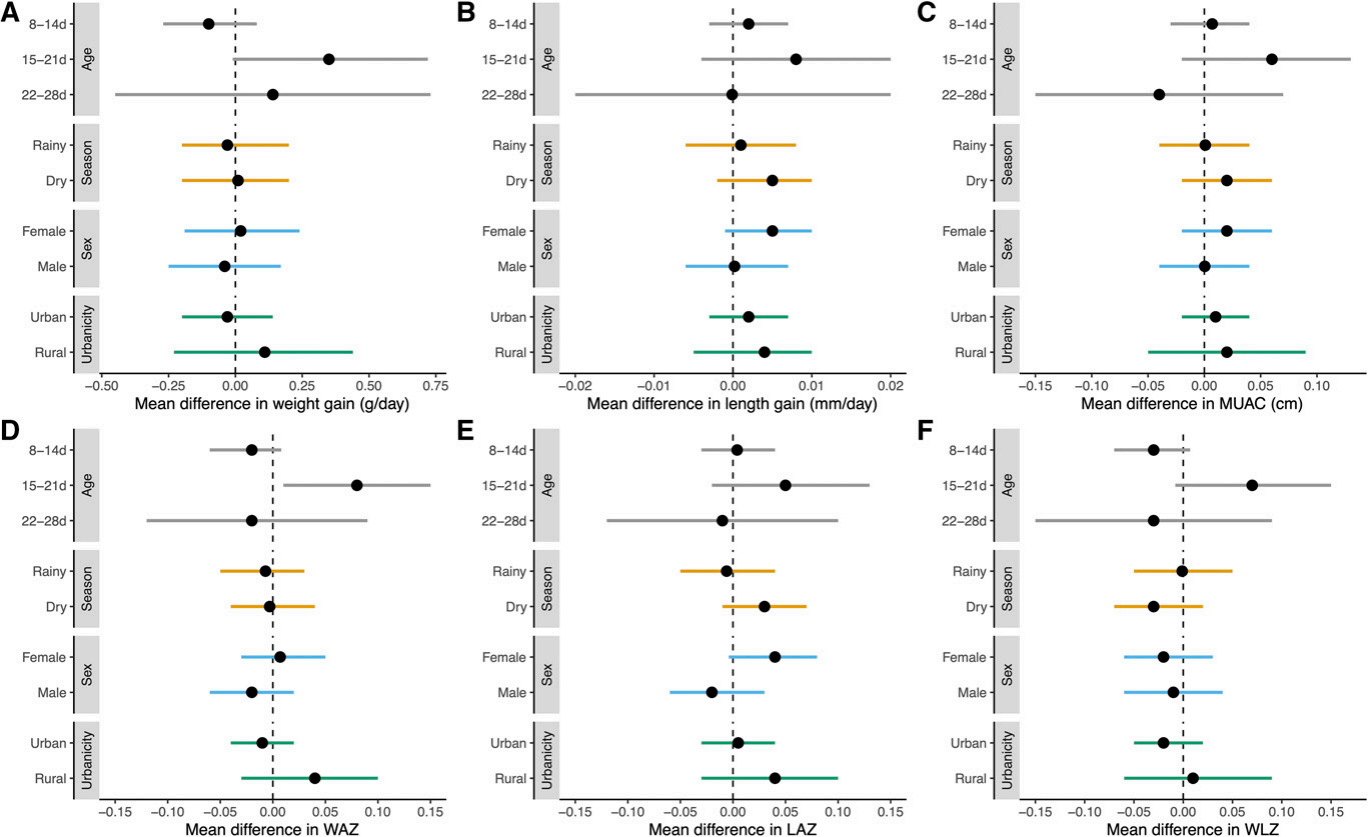
Mean differences in subgroups of neonates randomized to azithromycin vs. placebo in (**A**) weight gain (grams per day), (**B**) length gain (millimeters per day), (**C**) mid-upper-arm circumference (MUAC), (**D**) weight-for-age Z-score (WAZ), (**E**) length-for-age Z-score (LAZ), and (**F**) weight-for-length Z-score (WLZ). Subgroups are defined by age at enrollment (gray bars; 8–14 days of age, 15–21 days of age, 22–28 days of age), season of enrollment (yellow bars; rainy [June–October] or dry [November–May], sex (blue bars, male or female), and urbanicity (green bars, urban or rural).

In the subgroup of infants enrolled at age 15 to 21 days, those who received azithromycin had significantly greater WAZ scores at 6 months of age compared with placebo (Figure 2) and the interaction term for subgroup by week of age by treatment group was statistically significant (*P* for interaction = 0.02). However, the difference in WAZ in this subgroup was small (mean change 0.08 SD, 95% CI: 0.004–0.15). We found no evidence of a difference in other outcomes in subgroups of age (Supplemental Tables 1–6 and Figure 2). We found no evidence of a difference in anthropometric outcomes in prespecified subgroups defined by child’s sex, urbanicity (urban versus rural), or season of enrollment (rainy versus dry; Supplemental Tables 1–6 and Figure 2).

There was no evidence of a difference in dichotomized outcomes, including the odds of wasting (defined by WLZ < −2 SD), moderate acute malnutrition (MUAC < 12.5 cm), underweight (WAZ < −2 SD), or stunting (HAZ < −2 SD; Table 3).

**Table 3:** Wasting, underweight, and stunting by randomized treatment group among infants 6 months of age who received azithromycin or placebo between 8 and 27 days of age

Outcome	Azithromycin	Placebo	OR (95% CI)	*P* value
	*N* with outcome (%)	*N* with outcome (%)		
Wasted	531 (5.6%)	559 (5.8%)	0.96 (0.85–1.09)	0.55
(WLZ < −2)				
Moderate acute malnutrition	276 (2.9%)	314 (3.3%)	0.96 (0.84–1.10)	0.56
(MUAC < 12.5 cm)*				
Underweight	658 (6.9%)	652 (6.8%)	1.03 (0.92–1.15)	0.64
(WAZ < −2)				
Stunted	884 (9.3%)	869 (9.0%)	1.04 (0.94–1.14)	0.47
(LAZ < −2)				

CI = confidence interval; HAZ, height-for-age Z-score; LAZ = length-for-age Z-score; MUAC = mid-upper-arm circumference; OR = odds ratio; WAZ = weight-for-age Z-score; WLZ = weight-for-length Z-score.

* MUAC measurements available for 18,512 children at 6 months.

Adverse events, including serious adverse events, have been previously reported.^17^ Of note, there was one case of infantile hypertrophic pyloric stenosis, which resolved after surgery, and no cases of intussusception.

## DISCUSSION

We found no evidence to support a growth-promoting effect of azithromycin when administered to neonates by 6 months of age. The sample size was large, and the trial was powered to detect very small effect sizes. Previous studies have shown growth-promoting effects of antibiotics in a meta-analysis of randomized controlled trial of children >1 month of age, particularly for weight gain.^7^ Children enrolled in trials included in the meta-analysis typically had preexisting morbidity, most commonly malnutrition. These children may have been more likely to have coexisting enteric or other infection that was susceptible to antibiotics, which may have contributed to improved weight outcomes. In the present study, children had to be at least 2,500 g to participate due to concerns that lower-weight children might be more likely to develop infantile hypertrophic pyloric stenosis, which has been described in observational studies to be possibly related to macrolide use in neonates,^16^^,^^19^ which may have selected for healthier children being enrolled in the study. Infants enrolled in the present study may have less often had coexisting infection that may be susceptible to antibiotics. Previous trials and observational studies used different antibiotics (including metronidazole, cotrimoxazole, and amoxicillin) with longer treatment durations versus the single dose used in the present trial. It is possible that different antibiotic classes or different treatment durations may affect growth outcomes differently. However, this study builds on existing work by demonstrating that single dose azithromycin does not lead to weight gain when administered during the neonatal period to infants without established morbidity.

Some observational analyses have suggested that early-life antibiotic exposure may be linked to obesity in children in higher-income settings.^11^^,^^20^ Antibiotic exposure during the prenatal period and early infancy has been suggested to alter the infant gut microbiome and lead to increased adiposity.^21^ Others, however, have suggested that observational associations linking antibiotic exposure to childhood obesity are due to unmeasured confounding.^22^ The randomized placebo-controlled nature of the present study eliminates concerns related to unmeasured confounding and confounding by indication that are inherent in many observational analyses. With the large sample size resulting statistical power to detect very small effects and thus very precise estimates, these data are reassuring that single-dose azithromycin during the neonatal period does not lead to increased risk of weight gain during early infancy. Overall, these data do not support the hypothesis that early-life antibiotic exposure increases weight gain during infancy and suggest that previous observational study findings may be due to unmeasured confounding.

Some evidence has suggested that antibiotic exposure during the first week of life led to decreased growth compared with later administration.^23^ In this analysis, azithromycin increased weight gain slightly more among children enrolled and treated during the 3rd week of life compared with those in the 2nd or 4th weeks. However, absolute differences in weight gain were small and not statistically significant when stratified by week of age. Reasons for any difference in the efficacy of azithromycin on weight gain by age are unclear and may be attributable to chance due to multiple comparisons given that multiple subgroups were analyzed. The infant microbiome undergoes rapid change during this period, and it is possible that any effect of azithromycin on growth outcomes could be mediated by changes in the infant microbiome that change over time. However, the role of azithromycin distribution to older neonates and young infants remains unclear and could be investigated in future studies.

The use of antibiotics during early infancy in the absence of a clear clinical indication, as in studies for the prevention of childhood mortality, must be balanced with potential risk of adverse events. As previously mentioned, observational studies have suggested that early-life exposure to macrolides may increase risk of infantile hypertrophic pyloric stenosis. The parent trial found no evidence of an increased risk of pyloric stenosis.^17^ Any weight gain associated with antibiotic use could be a potential mechanism for previously observed effects of azithromycin on childhood survival, but also could be viewed as an adverse effect of antibiotic use if this predisposed children to obesity later in life. Results of the present study suggest both that growth promotion is not a major mechanism for the effect of azithromycin on childhood mortality and also that azithromycin-based interventions for childhood survival are unlikely to induce significant childhood obesity at the population level.

This study had some limitations. Previous evidence has suggested a short-term but no longer-term effect of azithromycin on weight gain in children aged 1 to 59 months.^24^ Anthropometric measurements were collected at baseline and 6 months of age and thus could have missed a short-term effect or longer term. However, any benefit of short-term but not longer-term improvements in weight gain in young infants for overall child health is unclear. We did not evaluate long-term effects of azithromycin on growth (e.g., past 6 months of age). Although it is possible that early disruptions to the gut microbiome could result in longer-term changes, we believe it is unlikely that we would observe longer-term effects of azithromycin on growth without evidence of a difference at 6 months. The dosing regimen was a single oral 20-mg/kg dose, consistent with what is used in trachoma control programs and in previous trials of azithromycin for prevention of childhood mortality. We did not record antibiotic prescriptions outside of the study between enrollment and 6 months of age. Previous analyses suggest that antibiotic prescription is common among children under 6 months of age in Burkina Faso.^25^^–^^27^ It is likely that many of the children enrolled in the present study received additional antibiotic prescription after enrollment, which could have made the two arms look more similar to one another and washed out any effect of a single dose of azithromycin during the neonatal period. However, we do not anticipate that antibiotic use is differential by arm, and these results suggest that a dose of azithromycin during the neonatal period does not affect growth outcomes by 6 months of age. We did not collect data on gut microbiome in this study and thus are unable to comment on how azithromycin may have altered the developing guts of infants in this study. Given that the gut microbiomes of neonates undergo rapid maturation during the first weeks of life, this is a priority for future research. Similarly, we did not evaluate selection for antibiotic resistance. Selection for macrolide resistance has been observed in studies of both individual and mass distribution of azithromycin for trachoma and childhood mortality, although any selection for macrolide resistance typically declines after selection pressure is removed.^28^^–^^30^ It is likely that the single dose of azithromycin led to short-term increases in macrolide resistance in the present study; however, any clinical implications of this are unclear. A companion trial to the present work found transient effects of azithromycin on pneumococcal macrolide resistance that returns to baseline levels by 6 months.^28^ The consequences of transient increases in macrolide resistance in potentially pathogenic organisms are not well characterized. Previous trials of mass azithromycin distribution have not found reductions in efficacy over time, suggesting that any selection for antimicrobial resistance does not reduce efficacy for preventing mortality, and macrolides are rarely used as first-line therapy in the study area.^31^^,^^32^ Additional research is needed to understand clinical outcomes in children with macrolide-resistant infections and the effect of azithromycin interventions on clinical outcomes in infections. Ongoing studies are evaluating the effect of single-dose azithromycin on selection for resistance in infants and will be reported separately. Many factors—including maternal characteristics, such as maternal nutritional status, infection, and socioeconomic status, and infant factors, including breastfeeding and exposure to infection—may influence growth. We did not measure some factors, such as improved water, sanitation, and hygiene, in this study, and nearly all infants (>99.9%) were exclusively breastfed, limiting the ability to evaluate differences in growth by these factors. However, the primary goal of this analysis was to understand the role of early-life antibiotic administration on infant growth. The randomized nature of the study ensures that there is no confounding by these or other factors because all exposures were randomized, and thus the study design is internally valid for evaluation of the core research question. This study did not collect data on costing, and thus we are unable to comment on cost implications of any azithromycin-based public health programming. Given that childhood growth is affected by nutrition, food sources and dietary diversity, and infectious disease burden, the results of this study may not be generalizable outside of similar West African settings.

We found no evidence that early life azithromycin distribution increases linear growth or weight gain in this randomized controlled trial of azithromycin compared with placebo administered to neonates aged 8 to 27 days of age. Although some observational analyses have suggested that early-life antibiotic administration may cause weight gain and predispose children to obesity, the results of the present study do not support that hypothesis. The use of azithromycin in child survival and trachoma programs is unlikely to contribute to childhood obesity, and growth promoting effects are unlikely to be a major contributor of azithromycin for improving infant survival.

## Financial Disclosure

The NAITRE study was funded by the Bill and Melinda Gates Foundation (OPP1187628). Azithromycin and matching placebo were donated by Pfizer, Inc (New York, NY). The funders had no role in study design, collection, analysis, or interpretation of data; in the writing of the report; or in the decision to publish.

## Supplemental Materials


Supplemental materials

## References

[1] Black RE , Maternal and Child Nutrition Study Group , 2013. Maternal and child undernutrition and overweight in low-income and middle-income countries. Lancet 382: 427–451. 23746772 10.1016/S0140-6736(13)60937-X

[2] Sudfeld CR , 2015. Malnutrition and its determinants are associated with suboptimal cognitive, communication, and motor development in Tanzanian children. J Nutr 145: 2705–2714. 26446481 10.3945/jn.115.215996

[3] Benjamin-Chung J , 2020. Early childhood linear growth failure in low- and middle-income countries. MedRxiv.

[4] Mertens A , 2020. Child wasting and concurrent stunting in low- and middle-income countries. MedRxiv. 10.1038/s41586-023-06480-z PMC10511327 37704720

[5] McEwen SA Fedorka-Cray PJ, 2002. Antimicrobial use and resistance in animals. Clin Infect Dis 34: S93–S106. 11988879 10.1086/340246

[6] Chattopadhyay MK, 2014. Use of antibiotics as feed additives: a burning question. Front Microbiol 5: 1–3. 25071747 10.3389/fmicb.2014.00334 PMC4078264

[7] Gough EK , 2014. The impact of antibiotics on growth in children in low and middle income countries: systematic review and meta-analysis of randomised controlled trials. BMJ 348: g2267. 24735883 10.1136/bmj.g2267 PMC3988318

[8] Aversa Z Atkinson EJ Schafer MJ Theiler RN Rocca WA Blaser MJ LeBrasseur NK, 2021. Association of infant antibiotic exposure with childhood health outcomes. Mayo Clin Proc 96: 66–77. 33208243 10.1016/j.mayocp.2020.07.019 PMC7796951

[9] Scott FI Horton DB Mamtani R Haynes K Goldberg DS Lee DY Lewis JD, 2016. Administration of antibiotics to children before age 2 years increases risk for childhood obesity. Gastroenterology 151: 120–129. 27003602 10.1053/j.gastro.2016.03.006 PMC4924569

[10] Johnston BC Srivastava A Chau K Kwon H Guo Q, 2020. Early and frequent exposure to antibiotics in children and the risk of obesity: systematic review and meta-analysis of observational studies. F1000 Res 9: 2020. 10.12688/f1000research.24553.1 PMC7429923 32913641

[11] Tian J Liu H Guo H Han W Ding H Chen T, 2020. Application of antibiotics before 3 years of age increases the risk of childhood overweight and obesity. Exp Ther Med 21: 1–7. 33273984 10.3892/etm.2020.9488 PMC7706393

[12] Trasande L Blustein J Liu M Corwin E Cox L Blaser M, 2013. Infant antibiotic exposures and early-life body mass. Int Obesity 37: 16–23. 22907693 10.1038/ijo.2012.132 PMC3798029

[13] Milliken S Allen RM Lamont RF, 2019. The role of antimicrobial treatment during pregnancy on the neonatal gut microbiome and the development of atopy, asthma, allergy and obesity in childhood. Expert Opin Drug Saf 18: 173–185. 30739516 10.1080/14740338.2019.1579795

[14] Keenan JD , 2018. Azithromycin to reduce childhood mortality in sub-Saharan Africa. N Engl J Med 378: 1583–1592. 29694816 10.1056/NEJMoa1715474 PMC5849140

[15] Oron AP , 2020. Effect modification by baseline mortality in the MORDOR Azithromycin Trial. Am J Trop Med Hyg 103: 1295–1300. 30734696 10.4269/ajtmh.18-1004 PMC7470539

[16] Sie A , 2019. Neonatal azithromycin administration to prevent infant mortality: study protocol for a randomised controlled trial. BMJ Open 9: e031162. 10.1136/bmjopen-2019-031162 PMC6731835 31488494

[17] Oldenburg CE , 2022. Neonatal azithromycin administration for prevention of infant mortality. NEJM Evidence 1: EVIDoa2100054. 35692260 10.1056/EVIDoa2100054 PMC9172759

[18] WHO Multicentre Growth Reference Study Group , 2006. WHO Child Growth Standards: Length/Height-for-Age, Weight-for-Age, Weight-for-Length, Weight-for-Height and Body Mass Index-for-Age: Methods and Development. Geneva, Switzerland: World Health Organization, 312.

[19] Eberly MD Eide MB Thompson JL Nylund CM, 2015. Azithromycin in early infancy and pyloric stenosis. Pediatrics 135: 483–488. 25687145 10.1542/peds.2014-2026 PMC9923582

[20] Baron R Taye M Besseling-Van Der Vaart I Ujčič-Voortman J Szajewska H Seidell JC Verhoeff A, 2020. The relationship of prenatal and infant antibiotic exposure with childhood overweight and obesity: a systematic review. J Dev Orig Health Dis 11: 335–349. 31735183 10.1017/S2040174419000722

[21] Zhang M Differding MK Benjamin-Neelon SE Østbye T Hoyo C Mueller NT, 2019. Association of prenatal antibiotics with measures of infant adiposity and the gut microbiome. Ann Clin Microbiol Antimicrob 18: 18. 31226994 10.1186/s12941-019-0318-9 PMC6587281

[22] Leong KSW , 2020. Associations of prenatal and childhood antibiotic exposure with obesity at age 4 years. JAMA Netw Open 3: e1919681. 31968118 10.1001/jamanetworkopen.2019.19681 PMC6991276

[23] Kamphorst K Oosterloo BC Vlieger AM Rutten NB Bunkers CM Wit EC Van Elburg RM, 2019. Antibiotic treatment in the first week of life impacts the growth trajectory in the first year of life in term infants. J Pediatr Gastroenterol Nutr 69: 131–136. 31058782 10.1097/MPG.0000000000002360

[24] Sié A , 2021. Single-dose azithromycin for child growth in Burkina Faso: a randomized controlled trial. BMC Pediatr 21: 130. 33731058 10.1186/s12887-021-02601-7 PMC7967941

[25] Sié A , 2019. Antibiotic prescription patterns among children younger than 5 years in Nouna District, Burkina Faso. Am J Trop Med Hyg 100: 1121–1124. 30693860 10.4269/ajtmh.18-0791 PMC6493961

[26] Sié A , 2021. Indication for antibiotic prescription among children attending primary healthcare services in rural Burkina Faso. Clin Infect Dis 73: 1288–1291. 34018004 10.1093/cid/ciab471 PMC8492132

[27] Oldenburg CE , 2021. Distance to primary care facilities and healthcare utilization for preschool children in rural northwestern Burkina Faso: results from a surveillance cohort. BMC Health Serv Res 21: 212. 33750364 10.1186/s12913-021-06226-5 PMC7941928

[28] Coulibaly B , 2022. Effect of single-dose azithromycin on pneumococcal carriage and resistance a randomized controlled trial. Pediatr Infect Dis J 41: 728–730. 35944061 10.1097/INF.0000000000003585 PMC9359759

[29] O’Brien K Emerson P Hooper PJ Dennis EG Keenan JD Lietman TM Oldenburg CE, 2018. Antimicrobial resistance following mass azithromycin distribution for trachoma: a systematic review. Lancet Infect Dis 19: e14–e25. 30292480 10.1016/S1473-3099(18)30444-4

[30] Doan T , 2020. Macrolide and nonmacrolide resistance with mass azithromycin distribution. N Engl J Med 383: 1941–1950. 33176084 10.1056/NEJMoa2002606 PMC7492079

[31] Keenan JD , 2019. Longer-term assessment of azithromycin for reducing childhood mortality in Africa. N Engl J Med 380: 2207–2214. 31167050 10.1056/NEJMoa1817213 PMC6512890

[32] Sie A , 2019. Antibiotic prescriptions among children under age 5 in Nouna District, Burkina Faso. Am J Trop Med Hyg 100: 1121–1124. 30693860 10.4269/ajtmh.18-0791 PMC6493961

