# Supplementary material for: Neonatal Azithromycin Administration and Growth during Infancy: A Randomized Controlled Trial
Source: Am J Trop Med Hyg. 2023 Mar 27;108(5):1063–70. doi: 10.4269/ajtmh.22-0763 (PMC10160881; doi:10.4269/ajtmh.22-0763)
Supplement: Supplementary file 1 [file tpmd220763.SD1.pdf]

Supplemental material for "Neonatal azithromycin administration and growth during infancy: a randomized controlled trial"

Supplemental Table 1. Results of sub-group analyses of effect of azithromycin versus placebo on weight gain (g/day)

	**Azithromycin**		**Placebo**			
	**N**	**Mean (SD)**	**N**	**Mean (SD)**	**Mean Difference (95 % CI)**	**P-value[1]**
**Age at enrollment**						
8 to 14 days	7077	23.4 (5.2)	7278	23.5 (5.4)	-0.1 (-0.3 to 0.1)	0.09
15 to 21 days	1634	23.0 (5.3)	1571	22.6 (5.2)	0.4 (-0.01 to 0.7)	
22 to 28 days	764	22.4 (6.1)	777	22.3 (5.7)	0.1 (-0.5 to 0.7)	
**Child's sex**						
Female	4691	22.4 (5.2)	4757	22.4 (5.4)	0.02 (-0.2 to 0.2)	0.70
Male	4784	24.0 (5.3)	4870	24.1 (5.3)	-0.04 (-0.3 to 0.2)	
**Season of enrollment**						
Rainy (June-October)	4593	23.4 (5.2)	4697	23.4 (5.5)	-0.03 (-0.2 to 0.2)	0.79
Dry (November-May)	4882	23.1 (5.4)	4930	23.1 (5.3)	0.01 (-0.2 to 0.2)	
**Urbanicity**						
Urban	7746	23.3 (5.4)	7945	23.3 (5.5)	-0.03 (-0.2 to 0.1)	0.51
Rural	1723	23.0 (4.9)	1672	22.9 (5.0)	0.1 (-0.2 to 0.4)	

[1]P for interaction for subgroup category by randomized treatment assignment

Supplemental Table 2. Results of sub-group analyses of effect of azithromycin versus placebo on length gain (mm/day)

	Azithromycin		Placebo			
	**N**	**Mean (SD)**	**N**	**Mean (SD)**	**Mean Difference (95 % CI)**	**P-value[1]**
**Age at enrollment**						
8 to 14 days	7077	0.9 (0.2)	7278	0.9 (0.2)	0.002 (-0.003 to 0.007)	0.64
15 to 21 days	1634	0.9 (0.2)	1571	0.9 (0.2)	0.008 (-0.004 to 0.02)	
22 to 28 days	764	0.9 (0.2)	777	0.9 (0.2)	-0.001 (-0.02 to 0.02)	
**Child's sex**						
Female	4691	0.9 (0.2)	4754	0.9 (0.2)	0.005 (-0.001 to 0.01)	0.26
Male	4784	0.9 (0.2)	4869	0.9 (0.2)	0.0002 (-0.006 to 0.007)	
**Season of enrollment**						
Rainy (June-October)	4593	0.9 (0.2)	4695	0.9 (0.2)	0.001 (-0.006 to 0.008)	0.45
Dry (November-May)	4882	0.9 (0.2)	4928	0.9 (0.2)	0.005 (-0.002 to 0.01)	
**Urbanicity**						
Urban	7746	0.9 (0.2)	7942	0.9 (0.2)	0.002 (-0.003 to 0.007)	0.73
Rural	1723	0.9 (0.1)	1672	0.9 (0.1)	0.004 (-0.005 to 0.01)	

[1]P for interaction for subgroup category by randomized treatment assignment

Supplemental Table 3. Results of sub-group analyses of effect of azithromycin versus placebo on mid-upper arm circumference (cm)

	Azithromycin		Placebo			
	N	Mean (SD)	N	Mean (SD)	Mean Difference (95 % CI)	P-value[1]
**Age at enrollment**						
8 to 14 days	7039	14.1 (1.1)	7244	14.1 (1.1)	0.007 (-0.03 to 0.04)	0.34
15 to 21 days	1632	14.1 (1.2)	1566	14.0 (1.2)	0.06 (-0.02 to 0.1)	
22 to 28 days	763	14.0 (1.2)	776	14.1 (1.2)	-0.04 (-0.2 to 0.07)	
**Child's sex**						
Female	4671	13.9 (1.1)	4736	13.9 (1.1)	0.02 (-0.02 to 0.06)	0.55
Male	4763	14.2 (1.2)	4850	14.2 (1.1)	0.0004 (-0.04 to 0.04)	
**Season of enrollment**						
Rainy (June-October)	4552	14.1 (1.2)	4658	14.1 (1.1)	0.0008 (-0.04 to 0.04)	0.49
Dry (November-May)	4882	14.0 (1.1)	4928	14.0 (1.1)	0.02 (-0.02 to 0.06)	
**Urbanicity**						
Urban	7709	14.1 (1.2)	7911	14.1 (1.2)	0.005 (-0.03 to 0.04)	0.67
Rural	1723	13.9 (1.1)	1672	13.8 (1.1)	0.04 (-0.03 to 0.1)	

[1]P for interaction for subgroup category by randomized treatment assignment

Supplemental Table 4. Results of sub-group analyses of effect of azithromycin versus placebo on weight-for-age Z-score

	Azithromycin		Placebo			
	**N**	**Mean (SD)**	**N**	**Mean (SD)**	**Mean Difference (95 % CI)**	**P-value[1]**
**Age at enrollment**						
8 to 14 days	7077	-0.42 (1.0)	7278	-0.40 (1.0)	-0.02 (-0.06 to 0.008)	0.02
15 to 21 days	1634	-0.45 (1.0)	1571	-0.54 (1.1)	0.08 (0.01 to 0.15)	
22 to 28 days	764	-0.62 (1.2)	777	-0.58 (1.1)	-0.02 (-0.12 to 0.09)	
**Child's sex**						
Female	4691	-0.32 (1.0)	4756	-0.32 (1.0)	0.007 (-0.03 to 0.05)	0.37
Male	4784	-0.57 (1.1)	4870	-0.54 (1.1)	-0.02 (-0.06 to 0.02)	
**Season of enrollment**						
Rainy (June-October)	4593	-0.42 (1.0)	4697	-0.40 (1.0)	-0.007 (-0.05 to 0.03)	0.92
Dry (November-May)	4882	-0.47 (1.1)	4929	-0.46 (1.1)	-0.003 (-0.04 to 0.04)	
**Urbanicity**						
Urban	7746	-0.42 (1.1)	7945	-0.40 (1.1)	-0.01 (-0.04 to 0.02)	0.22
Rural	1723	-0.55 (1.0)	1672	-0.58 (1.0)	0.04 (-0.03 to 0.1)	

[1]P for interaction for subgroup category by randomized treatment assignment

Supplemental Table 5. Results of sub-group analyses of effect of azithromycin versus placebo on length-for-age Z-score

	Azithromycin		Placebo			
	**N**	**Mean (SD)**	**N**	**Mean (SD)**	**Mean Difference (95 % CI)**	**P-value[1]**
**Age at enrollment**						
8 to 14 days	7077	-0.45 (1.2)	7278	-0.44 (1.1)	0.004 (-0.03 to 0.04)	0.45
15 to 21 days	1634	-0.48 (1.1)	1571	-0.53 (1.1)	0.05 (-0.02 to 0.1)	
22 to 28 days	764	-0.59 (1.2)	777	-0.57 (1.2)	-0.01 (-0.1 to 0.1)	
**Child's sex**						
Female	4691	-0.26 (1.1)	4754	-0.28 (1.1)	0.04 (-0.004 to 0.08)	0.07
Male	4784	-0.67 (1.2)	4869	-0.64 (1.2)	-0.02 (-0.06 to 0.03)	
**Season of enrollment**						
Rainy (June-October)	4593	-0.48 (1.2)	4695	-0.45 (1.2)	-0.006 (-0.05 to 0.04)	0.29
Dry (November-May)	4882	-0.45 (1.2)	4928	-0.47 (1.1)	0.03 (-0.01 to 0.07)	
**Urbanicity**						
Urban	7746	-0.48 (1.2)	7942	-0.48 (1.2)	0.005 (-0.03 to 0.04)	0.45
Rural	1723	-0.39 (1.1)	1672	-0.41 (1.1)	0.04 (-0.03 to 0.1)	

[1]P for interaction for subgroup category by randomized treatment assignment

Supplemental Table 6. Results of sub-group analyses of effect of azithromycin versus placebo on weight-for-length Z-score

	Azithromycin		Placebo			
	**N**	**Mean (SD)**	**N**	**Mean (SD)**	**Mean Difference (95 % CI)**	**P-value[1]**
**Age at enrollment**						
8 to 14 days	1625	-0.12 (1.2)	1569	-0.09 (1.2)	-0.03 (-0.07 to 0.007)	0.07
15 to 21 days	761	-0.12 (1.2)	772	-0.20 (1.2)	0.07 (-0.008 to 0.15)	
22 to 28 days	7061	-0.26 (1.2)	7263	-0.21 (1.2)	-0.03 (-0.15 to 0.09)	
**Child's sex**						
Female	4672	-0.12 (1.2)	4743	-0.10 (1.2)	-0.02 (-0.06 to 0.03)	0.84
Male	4775	-0.14 (1.2)	4861	-0.13 (1.2)	-0.01 (-0.06 to 0.04)	
**Season of enrollment**						
Rainy (June-October)	4577	-0.08 (1.2)	4685	-0.08 (1.2)	-0.001 (-0.05 to 0.05)	0.46
Dry (November-May)	4870	-0.18 (1.2)	4919	-0.15 (1.2)	-0.03 (-0.07 to 0.02)	
**Urbanicity**						
Urban	7720	-0.08 (1.2)	7925	-0.07 (1.2)	-0.02 (-0.05 to 0.02)	0.49
Rural	1722	-0.34 (1.1)	1670	-0.35 (1.1)	0.01 (-0.06 to 0.09)	

[1]P for interaction for subgroup category by randomized treatment assignment
